# Fibroblast Migration in 3D is Controlled by Haptotaxis in a Non-muscle Myosin II-Dependent Manner

**DOI:** 10.1007/s10439-015-1343-2

**Published:** 2015-05-27

**Authors:** O. Moreno-Arotzena, C. Borau, N. Movilla, M. Vicente-Manzanares, J. M. García-Aznar

**Affiliations:** Multiscale in Mechanical and Biological Engineering (M2BE), Department of Mechanical Engineering, Aragon Institute of Engineering Research (I3A), University of Zaragoza, 50018 Saragossa, Spain; Department of Medicine, Hospital Universitario de la Princesa, Universidad Autonoma de Madrid School of Medicine, 28006 Madrid, Spain

**Keywords:** Microfluidics, Hydrogel, Collagen, Fibrin, Chemotaxis, Mechanical properties

## Abstract

Cell migration in 3D is a key process in many physiological and pathological processes. Although valuable knowledge has been accumulated through analysis of various 2D models, some of these insights are not directly applicable to migration in 3D. In this study, we have confined biomimetic hydrogels within microfluidic platforms in the presence of a chemoattractant (platelet-derived growth factor-BB). We have characterized the migratory responses of human fibroblasts within them, particularly focusing on the role of non-muscle myosin II. Our results indicate a prominent role for myosin II in the integration of chemotactic and haptotactic migratory responses of fibroblasts in 3D confined environments.

**Electronic supplementary material:**

The online version of this article (doi:10.1007/s10439-015-1343-2) contains supplementary material, which is available to authorized users.

## Introduction

Cellular migration is a central event in physiological and pathological processes. Individual cell migration has been extensively characterized in two-dimensional (2D) models,^47^ and these approaches have yielded most of our current knowledge on the molecular regulation of the component processes of cell migration, i.e., polarization, protrusion, adhesion, displacement of the cell body and retraction. However, cell migration *in vivo* is seldom 2D. Hence, cell migration is better addressed in three-dimensional (3D) conditions to resemble the real cellular microenvironment. In this regard, several studies have shown that the cellular mechanics and migratory mechanisms of the same cells are quite different in 2D and 3D.^3^

Cell migration through 3D interstitial tissues is a multi-step process. The extracellular matrix (ECM) constitutes a heterogeneous multi-cue microenvironment that directly affects cell behavior,^7^ playing a central role in processes such as wound healing or tumor metastasis. It provides architectural scaffolding and orchestrates biochemical and biomechanical cues. Cells sense the mechanical properties and convert them into biological responses through the cytoskeleton by initiating signaling cascades that, among other responses, exert traction forces.^18^,^23^ In this process, biochemical signals are also able to influence the mechanical sensing capability of the cell.^37^,^55^ The integration of mechanical sensing and biochemical activation determines the ability of cells to migrate, their phenotype and their ability to remodel the matrix as they migrate.^39^

The capability of cells to sense and respond to the environmental cues is complex and dynamic,^9^,^14^ and alterations in this balance participate in the onset of several pathologies.^6^,^45^ For instance, in acute wound healing, the contraction level is regulated by the cells through growth factors (GFs) and rigidity-sensing mechanisms, coordinating the healing process.^46^ Furthermore, fibroblast differentiation into myofibroblasts—the contractile phenotype—enables the final closure of the wound and drives locally continuous stiffening, leading to the assembly of fibrotic tissue.^45^

Currently, various natural self-assembling ECM proteins are used to construct biomimetic hydrogels to perform *in vitro* studies.^51^ However, the combination of mechanical and biochemical properties of these gels drastically determine the migratory ability of the embedded cells,^29^,^30^,^53^ making it essential to thoroughly characterize these properties to decouple their individual contributions to the cellular migratory response.

3D cell migration depends on the physico-chemical balance between cell deformability and physical tissue constraints,^52^ both depending on ligand density, cross-linking level and architecture.^20^ Ligand density correlates with binding sites for integrin receptors. Cross-linking concentration determines the susceptibility of the network to degradation by proteolytic enzymes and the fibrillar 3D arrangement—porosity, pore size and fiber diameter-,^13^ thus, critically controlling the stiffness of the gel.^4^,^51^ The microstructure determines the permeability of the matrix, which directs the transport of biomolecules and local hydraulic asymmetries in the cell surroundings.^9^ Together, all these parameters critically control cell migration: ligand density,^49^ stiffness,^25^,^53^ microstructure,^12^,^17^ local permeability gradients 33,44 and external loading.^32^,^40^

In 3D, some of the biochemical cues that enable cell migration are immobilized in the matrix, whereas others diffuse through the meshwork. For example, GFs, chemokines and other biomolecules diffuse through the pores of the network forming chemical gradients. The matrix may act not only as a diffusion controller through pore size and connectivity, but also as a factor-reservoir, by providing available binding sites to the biomolecules.^22^,^26^ Based on this, chemical gradients at the microscopic level are heterogeneous in a context-dependent manner.^9^ They may get bound or remain as soluble factors and have a distinct role regulating cell migration.

Microfluidics enables precise control of this microenvironmental complexity. It also offers versatility for a rational design of the experiments—by defining biochemical and biomechanical cues- and real-time visualization—allowing *in vivo* quantification. Due to all these advantages, the use of microfluidic platforms is on the rise for studying 3D migration,^31^ including angiogenesis,^5^ metastasis^54^ and neuronal migration assays.^28^

Another key example of relevant migratory phenomenon is wound healing. Several studies have addressed fibroblast mechanics, growth factor (GF) signaling and matrix remodeling.^11^,^24^,^34^ These approaches have also addressed the role of multiple spatial cues, requirement for integrin-dependent adhesion and the assembly of actin-based structures.^2^,^42^ Responses have turned out to be context-dependent, by adapting dynamically the migration mode to local architecture and proteolytic and mechanical properties.^7^,^50^

To the best of our knowledge, 3D fibroblast migration has not been studied by using microfluidic devices. In this work, two physiologically relevant matrices have been characterized, and their ability to support fibroblast migration analyzed in a highly quantitative manner. We have used collagen I and fibrin matrices due to their crucial role in different phases of wound healing. Fibrin is the main constituent of the matrix during clotting, whereas collagen I is synthesized and remodeled by migrating fibroblasts to form the scar. The hydrogels were injected and confined into the microdevices to mimic confined processes such as granulation^19^ and connective tissue remodeling.^45^

Initially, we have characterized the biophysical properties of each matrix, followed by quantification of fibroblast migration in the two matrices in response to chemotactic stimulation with platelet-derived growth factor-BB (PDGF-BB). Finally, we have addressed the role of non-muscle myosin II (NMII) in fibroblast migration under these conditions. We have concluded that fibroblast migration is critically controlled in a NMII-dependent manner. Our results indicate that, although chemotactic and haptotactic signals enhance directional migration, they are not sufficient by themselves to overcome the restrictions imposed by the lack of functional myosin II in live cells.

## Materials and Methods

### Microfluidic Platform

**Figure 1:**
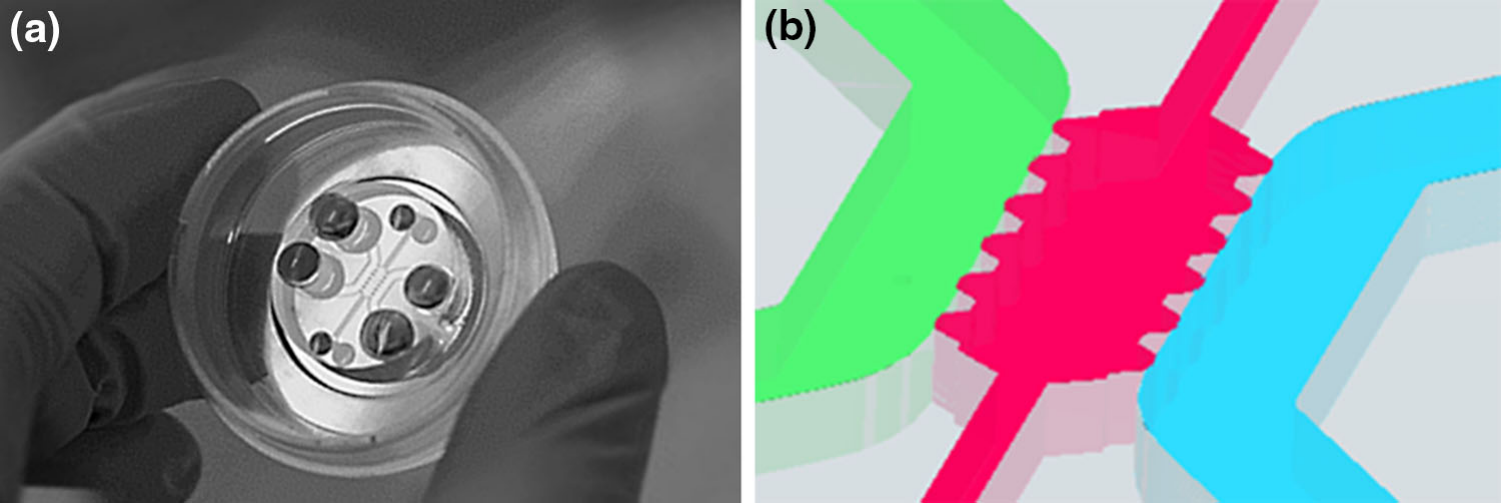
Microfluidic platform. The microdevice fits within a 35 mm glass-bottom petri dish, as shown in picture (a). The detailed schematic (b) shows the geometry of the microfluidic device. The hydrogel is kept confined within the central channel (pink), whose dimensions are 2.5 × 1.3 mm. The auxiliary channels (pink) assist the hydrogel injection into the central cage. In direct contact to the gel, two main media channels (1 mm-width, green and blue) ensure hydration and diffusion through the hydrogel. The height of the channels is of 240 *μ*m all over the geometry.

Microdevices were carried out following the methodology described by Shin *et al.*^41^ Hence, soft lithography was employed to achieve positive SU8 240 *μ*m-relief patterns of the desired geometry onto a silicon wafer (Stanford University). Polydimethylsiloxane (PDMS, Sylgard 184, Dow Corning GmbH), mixed at a 10:1 ratio of base to curing agent, was then poured and cured onto the SU8 master. The replica-molded layer was trimmed, perforated and autoclaved. Finally, the PDMS devices and 35 mm glass-bottom petri dishes (Ibidi) were plasma bonded and treated with poly-d-lysine (PDL) solution at 1 mg mL^−1^ (Sigma-Aldrich) for an enhanced surface-matrix attachment (see Fig. 1a).

The geometry of the device was based on that used by Farahat *et al.*,^5^ as shown in Fig. 1b. It comprised a central cage which contained the hydrogel with the embedded cells. In direct contact to the gel, it also had two media channels in order to ensure hydration and transport of nutrients and other chemical factors throughout the hydrogel.

### Hydrogel Preparation and Cell Seeding

#### Cell Culture

Normal human dermal fibroblasts (NHDF, Lonza) were cultured up to passage 10 using Fibroblast Growth Medium-2 (FGM-2, Lonza). The cells were passaged or used for experiments when they reached 80% of confluence. Hydrogels were loaded with cells harvested by sequential trypsinization and centrifugation, and mixed with the gel solutions at a final concentration of 0.5 × 10^6^ cells mL^−1^.

#### Collagen Solution

We followed the procedure described by Shin *et al.*^41^ Briefly, collagen type I (BD Biosciences) was buffered to a final concentration of 2 mg mL^−1^ with 10× DPBS—calcium, magnesium—(Gibco), cell culture grade water (Lonza) and the cell solution. The dilution was brought to pH 7.4 with NaOH.

#### Fibrin Solution

Human Fibrinogen—plasminogen, fibronectin, factor XIII depleted—was mixed, to a final concentration of 3.3 mg mL^−1^, with Human Factor XIII (22 *μ*g mL^−1^) and human alpha-thrombin (1 U mL^−1^), from American Diagnostica GmbH. CaCl_2_ (5 mM, Sigma) and the cell solution were added to the mixture, with a final pH of 7.4.

#### Hydrogel Polymerization

As soon as the gel solution was ready, it was pipetted into the gel cavity using the auxiliary channels (see Fig. 1). Upon insertion, the samples were allowed to polymerize inside humid chambers. The gels were then hydrated with FGM-2 and stored in the incubator for 24 h before initiating the experiments, to ensure stabilization of the matrix and cell adhesion and conditioning.

### Microstructural and Rheology Studies

An integral biophysical and biomechanical characterization of the hydrogels was performed in a previous work.^27^ Microstructural analysis was carried out using scanning electron microscopy and confocal reflection imaging analysis. The resistance to flow of the gels was also assessed by quantifying their permeability or hydraulic conductivity. Finally, oscillatory strain amplitude sweeps were performed using a rheometer and the elastic and viscous shear moduli were measured. For each hydrogel, three independent samples were studied and data are presented as the mean ± SEM.

### Chemical Conditioning

After 24 h of incubation since polymerization, the 3D systems were ready to use. As controls, culture media (without any growth factor) was renewed in both media channels. In inhibition experiments, medium in both channels contained 30 *μ*M (±) blebbistatin (diluted in DMSO, EMD Millipore) or vehicle control (DMSO, Amresco), respectively.

The establishment of PDGF-BB (Abcam) gradient across the gel was achieved by adding the GF containing culture media (5 ng mL^−1^) to only one channel (green), while new medium alone was added to the other channel (blue) (refer to Fig. 1b). Thus, the chemical gradient was established by a diffusive process across the hydrogel.

The spatial distribution of PDGF-BB chemical gradient in both collagen and fibrin hydrogels was predicted by numerical simulations. As detailed in a previous work,^26^ a computer framework was developed based on a reaction–diffusion transport model, which was experimentally validated by enzyme-linked immunosorbent assays (ELISAs). This mathematical approach is able to estimate diffusion and binding mechanism patterns yielded from an established chemical gradient through fibrous matrices.

### Immunofluorescence Staining and Imaging

The samples were stained for both vinculin and phalloidin and imaged using a Nikon D-Eclipse C1 Confocal Microscope—equipped with a Plan Apo VC 60XH objective- and an Olympus Fluoview FV10i Confocal Microscope—with an UPLSAPO 60XW objective. To do so, once the cells were fixed in 4% paraformaldehyde (Affymetrix) in PBS for 20 min at room temperature, samples were washed in PBS three times and permeabilized with 0.1% Triton X-100 (Calbiochem) in PBS at room temperature. Cells were washed another three times and blocked with 3% goat serum (Sigma) in 5% BSA/PBS solution for 4 h at room temperature. Afterwards, the devices were incubated overnight at 4 °C with mouse anti-human hVin-1 antibody (ab11194, Abcam) at 1:100 in 0.5% BSA/PBS. Then, after washing the samples five times with 0.5% BSA/PBS, incubation with Alexa Fluor^®^ 488 goat anti-mouse antibody (A11029, Molecular Probes) at 1:100 and the conjugated Alexa Fluor^®^ 594 phalloidin (A12381, Molecular Probes) at 1:200 was done for 3 h at room temperature in the dark. Finally, cells were washed three times with 0.5% BSA/PBS, two more times with PBS, and subsequently imaged.

### Cell Tracking

Once the chemical arrangement for each device was done, all the samples were allowed to warm up for 30 min. Then, time-lapse imaging was carried out by acquiring phase contrast images every 20 min for 24 h. The focal plane was chosen to be in the middle along the *z*-axis of the device. Cells that went out of focus were not quantified, assuming that they moved to the bottom or to the top part of the chip. It intended to minimize the edge effects resulting from the glass and PDMS surfaces by ensuring that the tracked cells were fully embedded within the 3D network. During the whole experiment, the incubation conditions were controlled and held at 37 °C, 5% of CO_2_ and 95% of humidity.

Approximately 150 cells were tracked out of each set of experimental samples. Cell trajectory acquisition was performed using a hand coded semi-automatic Matlab^®^ script. By comparison of pixel intensities and using matrix convolution techniques, the software was able to find and track cell centroids, requesting the user for visual correction, and finally post-processing the migration results. As for the measurements, the whole trajectories of each individual cell were tracked. Later on, trajectories were outlined in different colors depending on the final position of the cell within the gel, which was virtually divided in three zones (see Fig. 5b); being red for zone 1, green for zone 2 and blue for zone 3. In addition, polar histograms were employed to display the directionality of cell migration. Likewise, mean—referring to the instantaneous—and effective—as to the euclidean—cell speeds were quantified and demonstrated by means of boxplots. The effective velocity for each tracked cell was calculated by taking into account the Euclidian distance between the initial and last points of the cell trajectory. In addition, the mean speed was computed through the average of all instantaneous velocities calculated every 20 min. Statistical analysis was performed by applying the Wilcoxon Sign test.

## Results

### Biophysical and Biomechanical Cues

We initially characterized the biophysical features of collagen I and fibrin gels. As presented in Table 1, our results showed that, on average, pore size and permeability are twofold higher in collagen than in fibrin gels. Stiffness of the collagen scaffolds was approximately 20-fold lower than those made of fibrin; elastic shear moduli of roughly 15 and 300 Pa were measured, respectively. These experimental parameters were used to interpret cell migration of human dermal fibroblast in next sections.

**Table 1:** Biophysical and biomechanical properties of collagen and fibrin hydrogels.^27^

	Collagen	Fibrin
Pore size (*μ*m)	2.84 ± 0.94	1.69 ± 0.33
Darcy’s permeability (m^2^)	1.00 × 10^−12^	5.73 × 10^−13^
Elastic shear modulus (Pa)	15.62 ± 0.28	295.99 ± 12.98
Viscous shear modulus (Pa)	1.83 ± 0.12	7.10 ± 0.82

The data of pore size and elastic and viscous moduli are presented as mean ± SEM

### Cell Morphology

**Figure 2:**
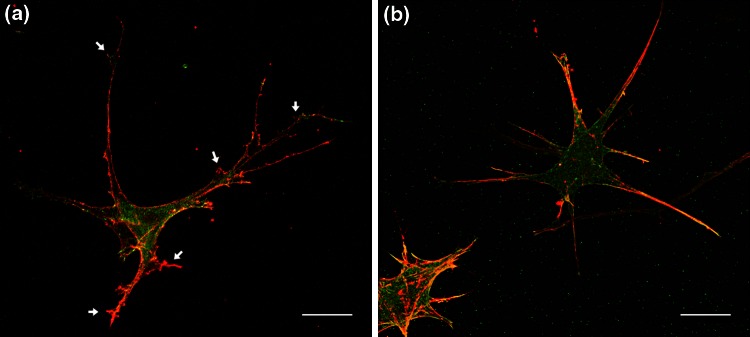
Fibroblast morphology in 3D by distribution of actin (red) and vinculin (green). The image in collagen (a) was taken by the Nikon D-Eclipse C1 Confocal Microscope and the one in fibrin (b) by employing the Olympus Fluoview FV10i Confocal Microscope. Cells in collagen appear stretched out, whereas in fibrin they are more frayed spindle-like. The white arrows point to some of the varicosities. Scale bars correspond to 20 *μ*m.

Fibroblasts seeded on collagen I and fibrin matrices displayed important differences in terms of cell shape and morphology. As shown in Fig. 2, fibroblasts in collagen gels were stretched out and displayed multiple, branched and long protruding structures with actin. Conversely, cells in fibrin hydrogels displayed frayed spindle-like protrusions and fewer actin patches in the projections.

### Quantitative Comparison of Spontaneous Fibroblast Migration in Collagen and Fibrin Hydrogels

**Figure 3:**
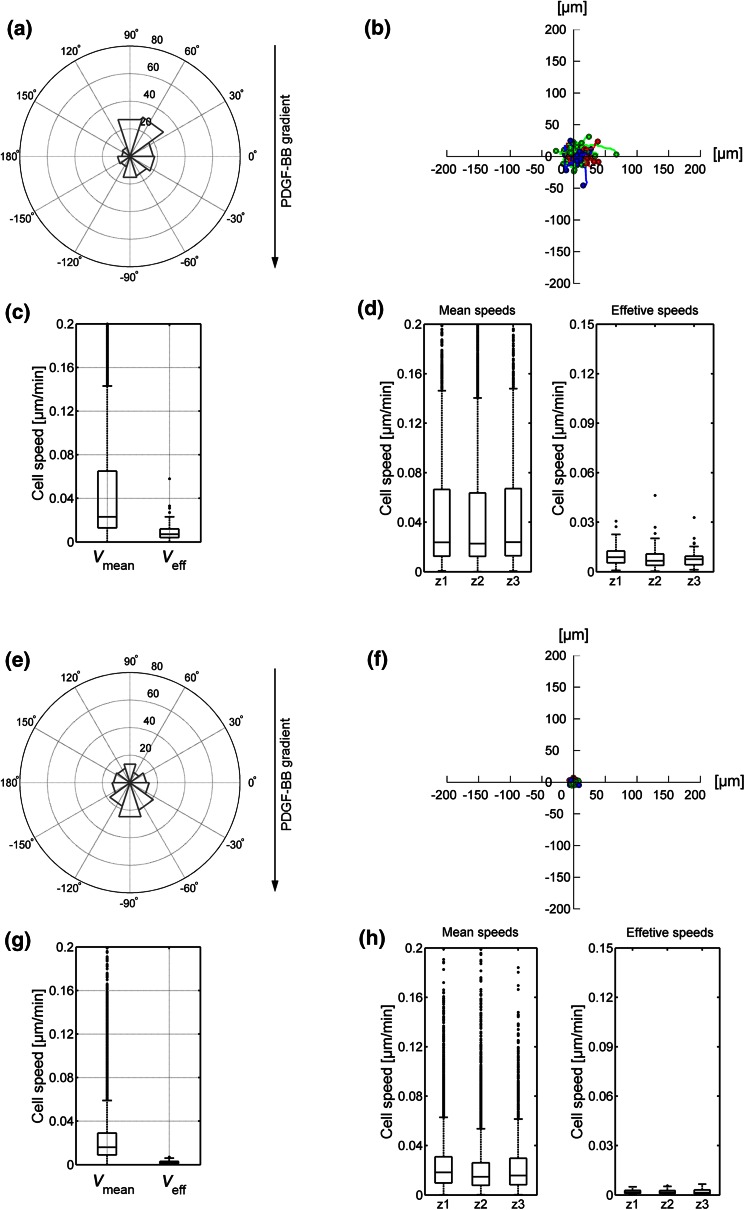
Migration quantification in control collagen (a–d) and fibrin (e–h) gels. Polar histograms (a, e) show the directionality of cell migration and represent the angle formed by the Euclidian distance between the initial and last points of every cell trajectory, being 90° the direction of the settled gradient. The histogram bins correspond to 36° and their radius magnitude represents the number of cells (radial number) that ended within that angular range. The gradient direction is illustrated by the black arrow, whose origin corresponds to the gradient source. The trajectories of individual cells are outlined (b, f); colors indicate the zone of the microdevice in which cells were located in the last time step (red corresponding to zone 1, green to zone 2 and blue to zone 3, respectively). Boxplots show the mean and effective speed of cells considering the whole device (c, g) or distinguishing the zone they belong to (d, h). Additional statistical data corresponding to polar histograms and boxplots are shown in the Supplementary Data.

**Figure 4:**
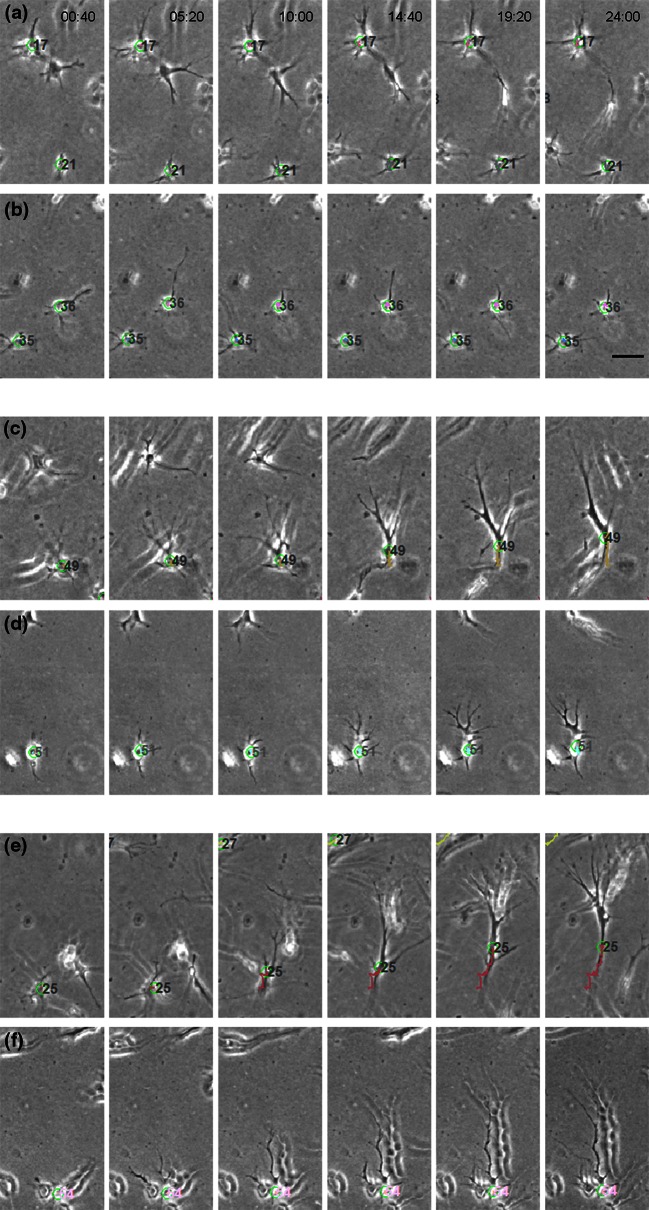
Sample time-lapse images of cells migrating in control samples (a, b), under PDGF-BB gradient (c, d) and under PDGF-BB gradient including blebbistatin (e, f). Samples correspond to fibroblast 3D migration in collagen (a, d, e) and fibrin (b, d, f) gels. Time points are indicated on the upper side of the image and the scale bar corresponds to 50 *μ*m. The number associated to the cells is irrelevant; in order to track the cells, it is automatically assigned and used only by the tracking program, accompanying the centroid (represented as a green circle).

When we compared the migratory behavior of dermal fibroblasts in both control gels, we observed important differences. Cells were less motile in fibrin. In the absence of a chemoattractant, cells did not persistently migrate in either matrix (Figs. 3a and 3b, 3e and 3f) and migratory speed was low in collagen (Figs. 3c and 3d), but even lower in fibrin (Figs. 3g and 3h). This was not due to an intrinsic inability of the cells to polarize or extend projections (Fig. 4). Interestingly, fibroblasts in collagen displayed robust “contractile shaking”, which was not observed in cells in fibrin gels (see SM1 and SM2).

### Characterization of PDGF-BB Gradients in Microfluidic Hydrogels

PDGF-BB is secreted by platelets during clotting and acts as a natural chemoattractant for dermal fibroblasts during wound healing. We took advantage of the intrinsic polarity of the microfluidic device to generate gradients of PDGF-BB and quantify the migratory properties of dermal fibroblasts as they navigate the hydrogels in its presence.

In a previous work^26^ we developed a computational tool, which was experimentally validated by enzyme-linked inmunosorbent assays (ELISAs), to assess transport and distribution of soluble GFs in 3D hydrogels. Actually, during transport, the biomolecules interact differently with the fibrillar network. Some of the biomolecules may degrade, diffuse through the pores or get bound to the matrix proteins. Knowing the proportion of these phenomena for a given GF–matrix combination is quite relevant to estimate cell migration, since it accounts for possible binding events that trigger haptotaxis (migration in response to immobilized factors) in addition to chemotaxis.

**Figure 5:**
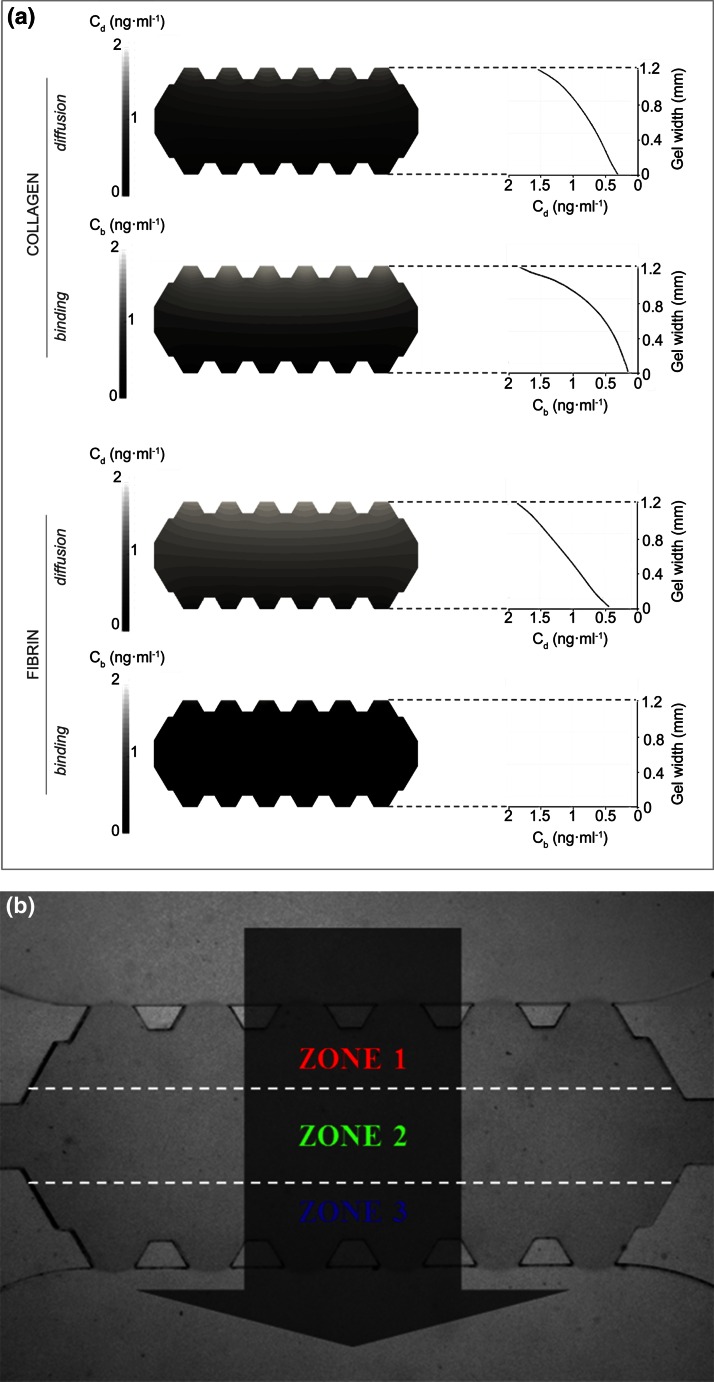
Spatial distribution of PDGF-BB in collagen and fibrin. Picture (a) shows the diffusion and binding concentration patterns yielded from the simulation of transport of the growth factor through collagen and fibrin hydrogels, after 24 h since gradient establishment at 5 ng mL^−1^. The graphs show quantitatively the evolution, over the gel width, of diffusion and binding concentration profiles. The concentration (ng mL^−1^) of the diffusing and binding growth factor is denoted as *C*_d_ and *C*_b_, respectively. Picture (b) is a widefield image of the gel region of the device, in which three zones are traced by the white dashed-lines for migration quantification. The dark arrow indicates the direction in which the chemical gradient is set; its origin denoting the maximum concentration. Trajectories corresponding to zone 1, 2 and 3 will be drawn in red, green and blue, respectively.

The computational tool, based on a reaction–diffusion model, is able to determine diffusion and binding processes that regulate the distribution and transport of chemical gradients through hydrogels.^26^ From there, the quantified spatio-temporal distribution of the GF inside both collagen and fibrin hydrogels is shown in Fig. 5a. In collagen gels, diffusion and binding events dominated the distribution of PDGF-BB inside the hydrogel. On the contrary, fibrin matrices displayed non-significant binding; hence, diffusion was the leading factor during its distribution in the hydrogel. There are accumulating data pointing out the physiological nature of specific GF to ECM-protein binding. In this regard, Somasundaram and Schuppan demonstrated the high affinity of PDGF-BB to bind collagen I.^43^ Conversely, the binding between PDGF-BB and fibrin has been determined to be of very short term,^22^ which explains the insignificant bound factor predicted by the simulation.

Based on this information, we defined three zones in the hydrogels (depicted in Fig. 5b). In collagen zone 1, effects are characterized by the strong effect of bound PDGF-BB, which decreases in zone 2 and 3. Soluble PDGF-BB would follow a linear distribution from the PDGF-BB-loaded channel. Conversely, binding is negligible in fibrin gels, hence the distribution of PDGF-BB is solely determined by a linear gradient stemming from the PDGF-BB-containing channel.

### Differential Effect of PDGF-BB on Cell Migration in Collagen and Fibrin Hydrogels

**Figure 6:**
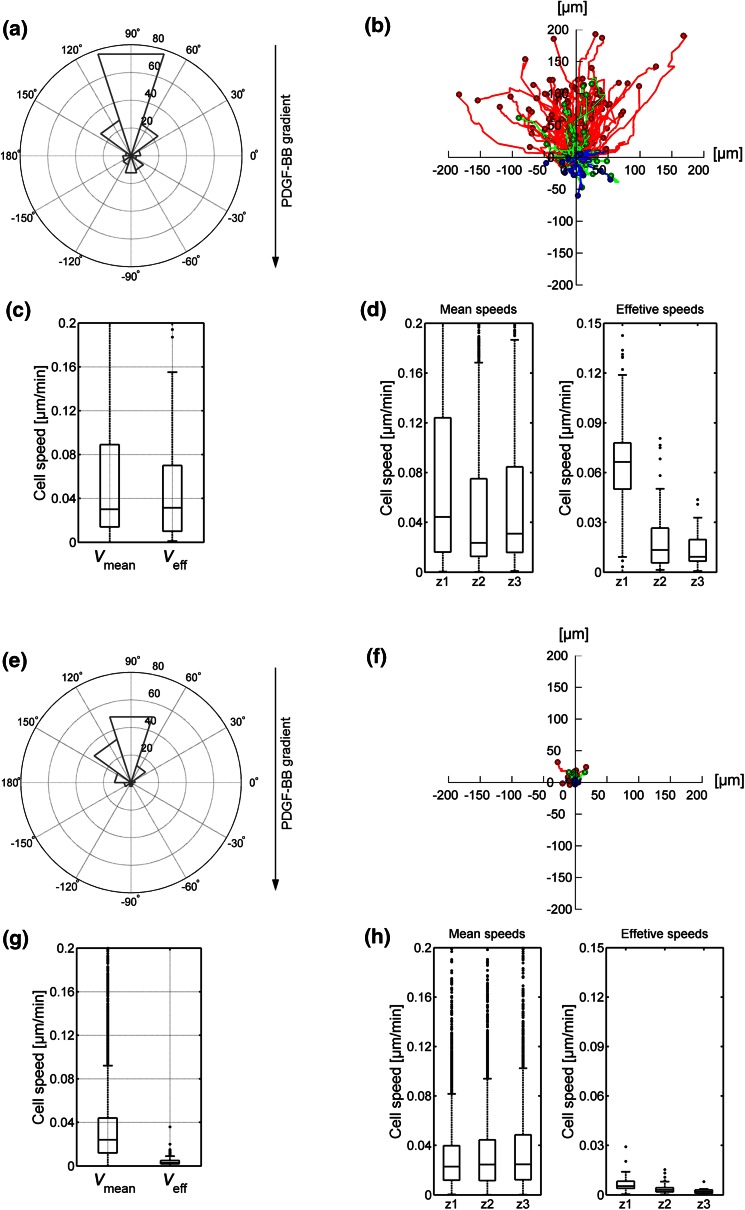
Migration quantification in PDGF-BB gradient-generated collagen (a–d) and fibrin (e–h) gels. Polar histograms (a, e) show the directionality of cell migration and represent the angle formed by the Euclidian distance between the initial and last points of every cell trajectory, being 90° the direction of the settled gradient. The histogram bins correspond to 36° and their radius magnitude represents the number of cells (radial number) that ended within that angular range. The gradient direction is illustrated by the black arrow, whose origin corresponds to the gradient source. The trajectories of individual cells are outlined (b, f); colors indicate the zone of the microdevice in which cells were located in the last time step (red corresponding to zone 1, green to zone 2 and blue to zone 3, respectively). Boxplots show the mean and effective speed of cells considering the whole device (c, g) or distinguishing the zone they belong to (d, h). Additional statistical data corresponding to polar histograms and boxplots are shown in the Supplementary Data.

We then sought to determine the effect of PDGF-BB on fibroblast migration in collagen and fibrin hydrogels. Embedded fibroblasts were exposed to PDGF-BB gradients and observed in collagen and fibrin gels (Figs. 4c and 4d, respectively). In both cases, cells exhibited an increased protrusiveness towards the source of PDGF-BB, as previously reported.^10^,^35^,^36^ However, protrusions were longer in cells within collagen (Supplemenatary Table S3). Increased protrusiveness correlated with increased motility (Fig. 6). The effect was much more significant in cells in collagen (compare Figs. 6b and 6f). Furthermore, cells within zone 1 displayed much higher speed than those in zone 2 (Fig. 6d). Comparatively, cells in zone 3 had no significant response to the gradient. Conversely, cells on fibrin displayed increased protrusiveness that resulted into a modest increase in cellular “wandering” (non-directional migration), but that effect did not translate into increased effective speed in any of the zones (Fig. 6h).

### Non-muscle Myosin II Controls Migratory Speed in Collagen Hydrogels

**Figure 7:**
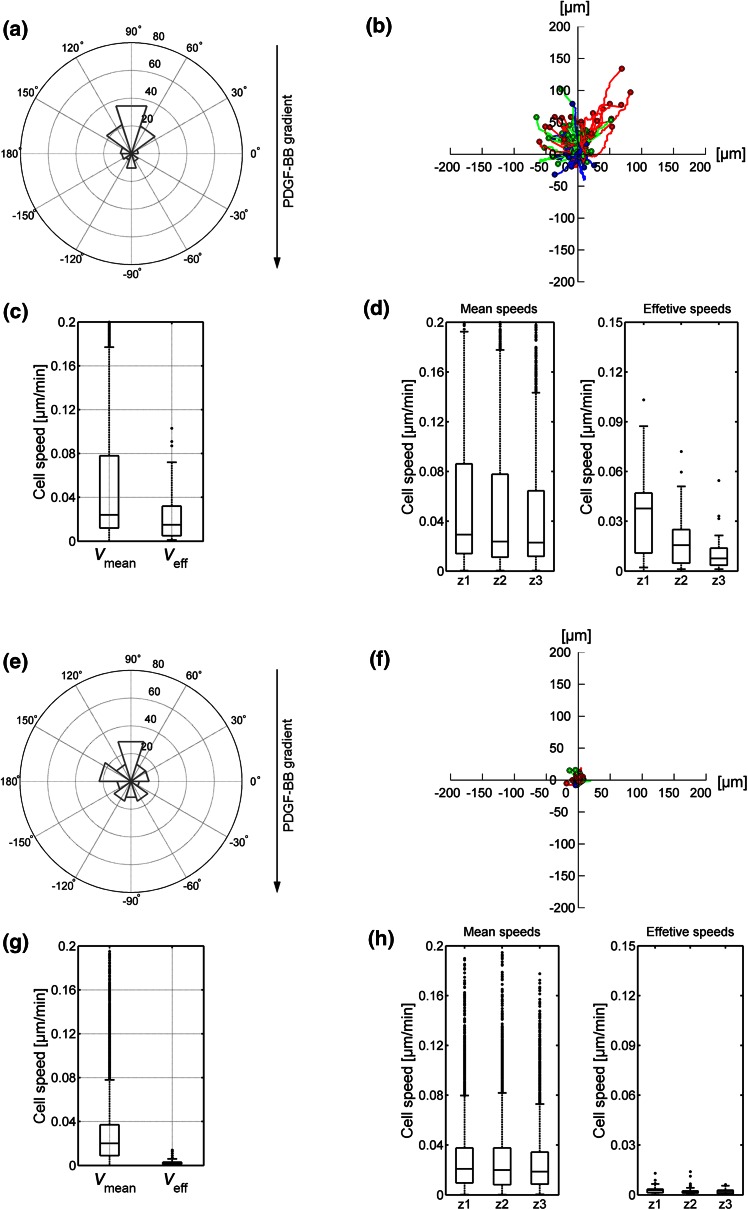
Migration quantification in PDGF-BB gradient-generated collagen (a–d) and fibrin (e–h) gels, including 30 µM blebbistatin. Polar histograms (a, e) show the directionality of cell migration and represent the angle formed by the Euclidian distance between the initial and last points of every cell trajectory, being 90° the direction of the settled gradient. The histogram bins correspond to 36° and their radius magnitude represents the number of cells (radial number) that ended within that angular range. The gradient direction is illustrated by the black arrow, whose origin corresponds to the gradient source. The trajectories of individual cells are outlined (b, f); colors indicate the zone of the microdevice in which cells were located in the last time step (red corresponding to zone 1, green to zone 2 and blue to zone 3, respectively). Boxplots show the mean and effective speed of cells considering the whole device (c, g) or distinguishing the zone they belong to (d, h). Additional statistical data corresponding to polar histograms and boxplots are shown in the Supplementary Data.

NMII modulates spontaneous fibroblast migration in 3D.^17^ To assess its role in directional 3D migration we infused the hydrogels with blebbistatin, which is a highly specific inhibitor of the ATPase activity of NMII, hence, blocking contractility.^16^ We found that, in fibrin, blebbistatin slightly increases protrusiveness, consistent with its effect in 2D,^47^ but this effect does not translate into increased migration (Figs. 7e–7h). In collagen, blebbistatin did not affect the orientation of the cells towards the gradient (Fig. 7a), or the emission of protrusions in the direction of the higher concentration of PDGF-BB (Fig. 4e). However, comparing to PDGF-BB samples, it attenuated migration towards PDGF-BB, particularly that of cells in zone 1 (Figs. 7b–7d). Together, these results suggest that NMII does not control the orientation of fibroblasts towards a chemotactic gradient in collagen hydrogels, but it does control the ability of cells to migrate efficiently.

## Discussion

In this work, we have combined microfluidics with hydrogels and gradients of soluble GFs in order to gain a better insight into fibroblast sensing and migratory mechanisms in 3D. For that, we used two biomimetic hydrogels, collagen and fibrin, characterized them and applied GFs to create a gradient and study fibroblast migration.

In general, our observations indicate that collagen gels promote fibroblast migration more efficiently than fibrin. In collagen, fibroblasts showed “contractile shaking”, likely due to cycles of protrusion extension and retraction as the cell explores its surroundings, “sensing” chemotactic and/or haptotactic cues. When such a signal is present (e.g., a PDGF gradient), the cell uses traction forces on the collagen fibers to establish front-rear polarity. As a consequence of polarity establishment, protrusion is mainly restricted to the leading edge. Cellular translocation occurs by the subsequent translation of the cell body. In this interpretation, extension and retraction forces are coordinately transmitted to the cell body to support forward motion. In fibrin, the cells show comparable extension and retraction of protrusions, meaning that the intrinsic actin polymerization (protrusion) and actomyosin contractility (retraction) is fully functional. However, the cell body does not move. This could be interpreted as lack of traction on fibrin, which would prevent transmission of the traction to the cell body for polarization and net movement. A related possibility is that, in fibrin, cells become less sensitive to chemical gradients, for example, by transmitting the gradient information poorly during the initiation of the front-rear polarization process. This is a major difference that likely underlies the different biochemical response observed when cells are confronted with a gradient of PDGF-BB in collagen or fibrin. Additional reasons may relate to biophysical issues such as pore size, permeability, the degree of polymer cross-linking and stiffness. Stiffness in collagen is 20-fold lower than in fibrin. However, it has been demonstrated that fibroblast migration in 3D is independent of matrix stiffness.^25^ Fibrin is more cross-linked than collagen, which decreases its susceptibility to degradation. Additionally, pore size and permeability in fibrin are approximately half the size of collagen gels. On the one hand, migration through small gaps has been shown to require proteolytic degradation of the matrix.^3^,^4^ In this context, the nucleus becomes a spatial hindrance for migration in the absence of degradation.^53^ On the other hand, confined migration has been shown to prefer environments with lower hydraulic resistance, even in chemotaxis-competing contexts.^33^ Actually, fibroblast migration has been pointed to be porosity-dependent.^25^ As to this interpretation, the narrower pore, reduced degradation and increased hydraulic resistance of the fibrin gels would impede productive migration in 3D. This points out that future systematic studies of the variation of the decoupled biomechanical properties of the matrix could potentially add new insights into the 3D cell migration.

Overall, cells were faster in collagen gels in response to PDGF-BB gradients, comparing to control samples, which was not surprising. However, segmentation of the migratory behavior of the cells with respect to the origin of the gradient revealed a non-linear response in terms of speed. Closer to the origin of the gradient (zone 1), cells showed a significant increase in effective speed, comparing to zone 1 cells in control samples. As for the medians, the increase in zone 1 was of eightfold, whereas it was only twofold in zone 2, i.e., more removed from the origin of the gradient. Cells in zone 3 (far from the origin) showed no significant increase in speed, for a similar comparison. The most obvious interpretation relies on the difference of diffusion-based PDGF-BB concentration between zones 1 and 2 (zone 1 is closer to the source). However, this would likely imply an almost linear difference between zones, which is not the case. Hence, some factor contributes to amplify the difference between zones 1 and 2. We noted that in collagen I zone 1, two populations of PDGF-BB appeared: one followed a diffusion phenomenon, whereas the other was immobile. We hypothesize that this second population was adsorbed or otherwise immobilized on collagen fibers, constituting a potent haptotactic signal. Several studies using EGF have demonstrated that immobilized GFs modify their properties towards inducing cell migration.^15^ This is likely due to increased signaling due to clustering of the receptor. An additional possibility is that PDGF-BB enhances integrin-mediated adhesion through a cross-talk mechanism.^38^

The decrease in cell migration due to NMII inhibition may be due to a number of factors: one is that NMII controls nuclear repositioning in migrating cells.^8^ In 3D, emerging evidence indicates that the nucleus is the main steric hindrance towards productive migration. It is feasible that NMII-inhibited cells get their nuclei “stuck” in the pores and are unable to migrate forward. In this interpretation, exaggerated protrusiveness results from inefficient attempts to compensate increased nuclear drag. A similar possibility is based on the fact that NMII controls cellular reshaping in response to compression/dilation.^21^ Taking this into account, NMII inhibition would lead to the cell losing its ability to deform in response to spatial constraints. Additional possibilities include deficient adhesion assembly. A recent study has shown that NMII inhibition prevents adhesion enlargement in 3D.^17^ Even if adhesions assemble, they do not reach a threshold size to transmit traction to the cell body, resulting in the same phenotype caused by nuclear drag. This possibility is further supported by the more dramatic effect of blebbistatin in zone 1 cells compared to zone 2, which suggests that the inhibition mechanism is related to the haptotactic response to PDGF-BB in this region by a more active participation of NMII in the cellular response to immobilized than soluble GFs in 3D. Another possibility is that myosin II is controlling the position of the protrusion, i.e., preventing actin from polymerizing except in the direction of the gradient. In 2D, fibroblast-like cells use NMII-B to suppress protrusion at the trailing edge.^48^ In this context, lateral and tail NMII-dependent contractions define amoeboid cell migration in 3D.^1^

## Conclusions

Multiple 3D migration modes have been proposed in several previous works. The context-dependent phenomenon is established by a dynamic and interrelated physicochemical balance, which makes complex elucidating the underlying mechanisms. In this work, by employing microfluidic-based models, we quantified and compared cell migration in 3D. By analyzing the chemotactic and haptotactic response to PDGF-BB cues, as well as to NMII inhibition, we determined that the haptotactic cues induced through the 3D networks regulate migration in a NMII-dependent manner. Specifically, NMII does not control the orientation of fibroblasts towards a chemical gradient in hydrogels, but it does control the ability of cells to migrate efficiently.

## Electronic Supplementary Material

Supplementary material 1 (DOCX 1036 kb)

Supplementary material 1 (ZIP 2506 kb)
