# Supplementary material for: Fibroblast Migration in 3D is Controlled by Haptotaxis in a Non-muscle Myosin II-Dependent Manner
Source: Ann Biomed Eng. 2015 May 27;43(12):3025–39. doi: 10.1007/s10439-015-1343-2 (PMC4623072; doi:10.1007/s10439-015-1343-2)
Supplement: Supplementary file 1 — Supplementary material 1 (DOCX 1036 kb) [file 10439_2015_1343_MOESM1_ESM.docx]

**Appendix A. Supplementary data**

Supplementary figures


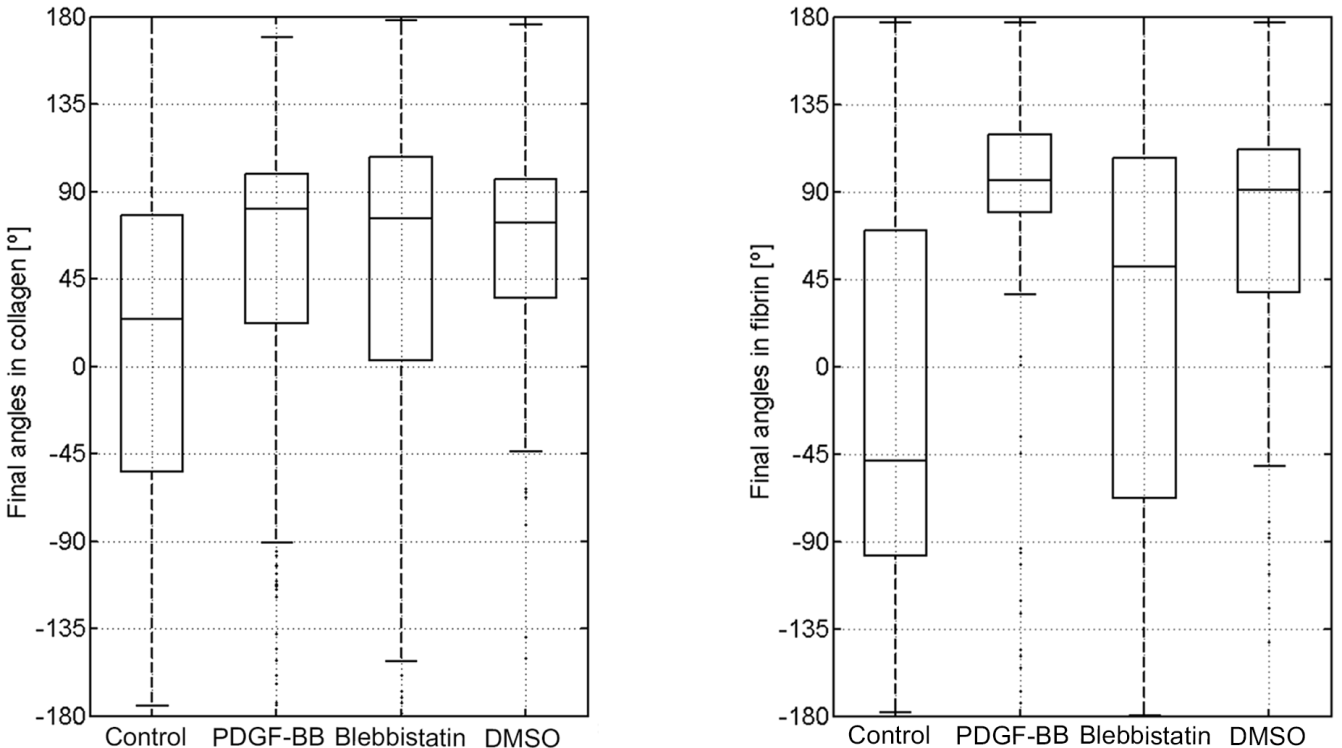


**Fig. S1.** Migration direction quantification. Directionality measurements of each cell in collagen (left) and fibrin (right) are shown in the boxplots, which correspond to data demonstrated by the polar histograms of Fig. 3, 6, 7 and S2. The corresponding median and (1st | 3rd) quartile values, along with the statistical significances, are gathered in Table S1.


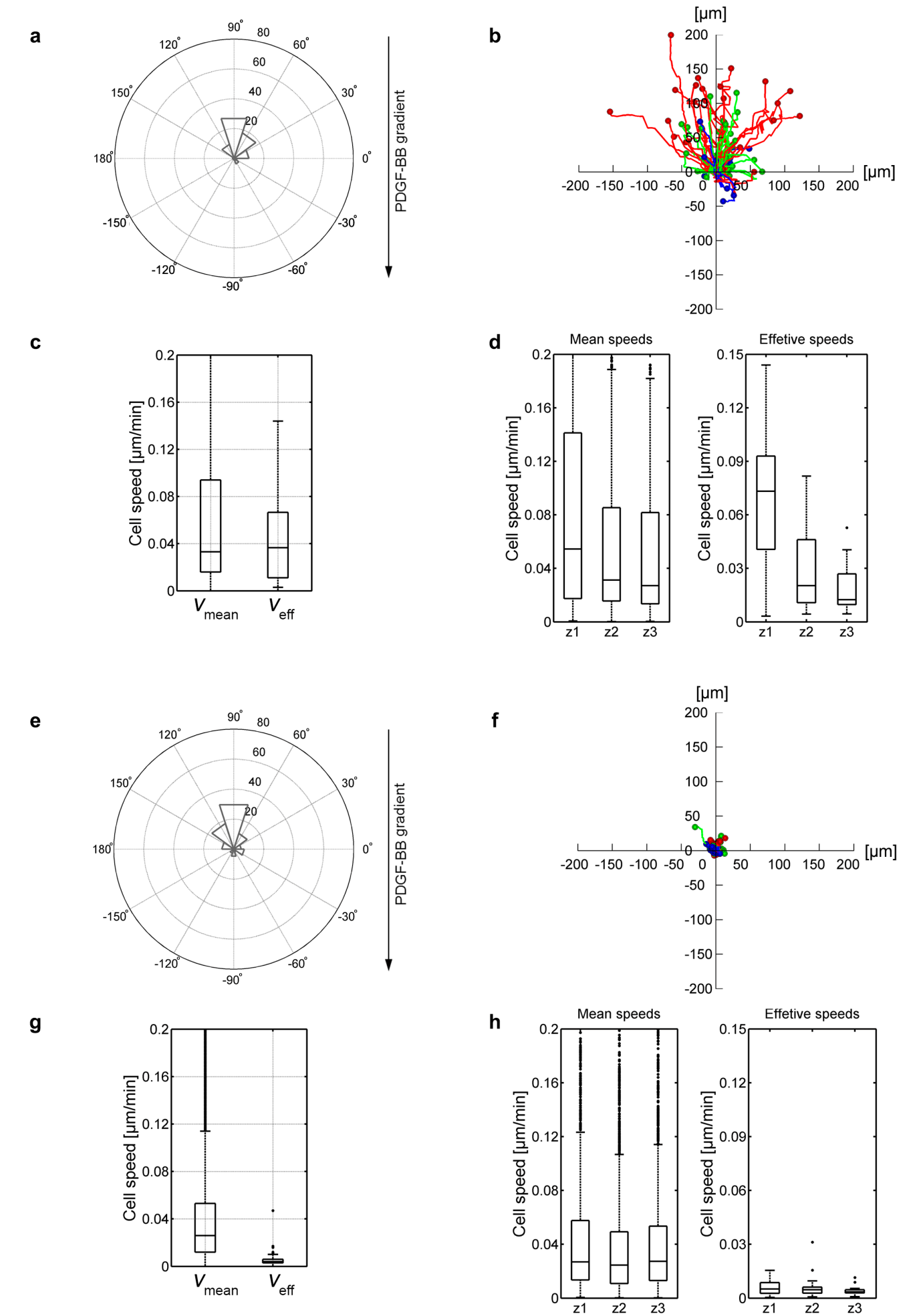


**Fig. S2.** Migration quantification in PDGF-BB gradient-generated collagen (a-d) and fibrin (e-h) gels, including vehicle-control. Polar histograms (a,e) show the directionality of cell migration and represent the angle formed by the Euclidian distance between the initial and last points of every cell trajectory, being 90º the direction of the settled gradient. The histogram bins correspond to 36º and their radius magnitude represents the number of cells (radial number) that ended within that angular range. The gradient direction is illustrated by the black arrow, whose origin corresponds to the gradient source. The trajectories of individual cells are outlined (b,f); colors indicate the zone of the microdevice in which cells were located in the last time step (red corresponding to zone 1, green to zone 2 and blue to zone 3, respectively). Boxplots show the mean and effective speed of cells considering the whole device (c,g) or distinguishing the zone they belong to (d,h). Additional statistical data corresponding to polar histograms and boxplots are shown in the Supplementary Data.


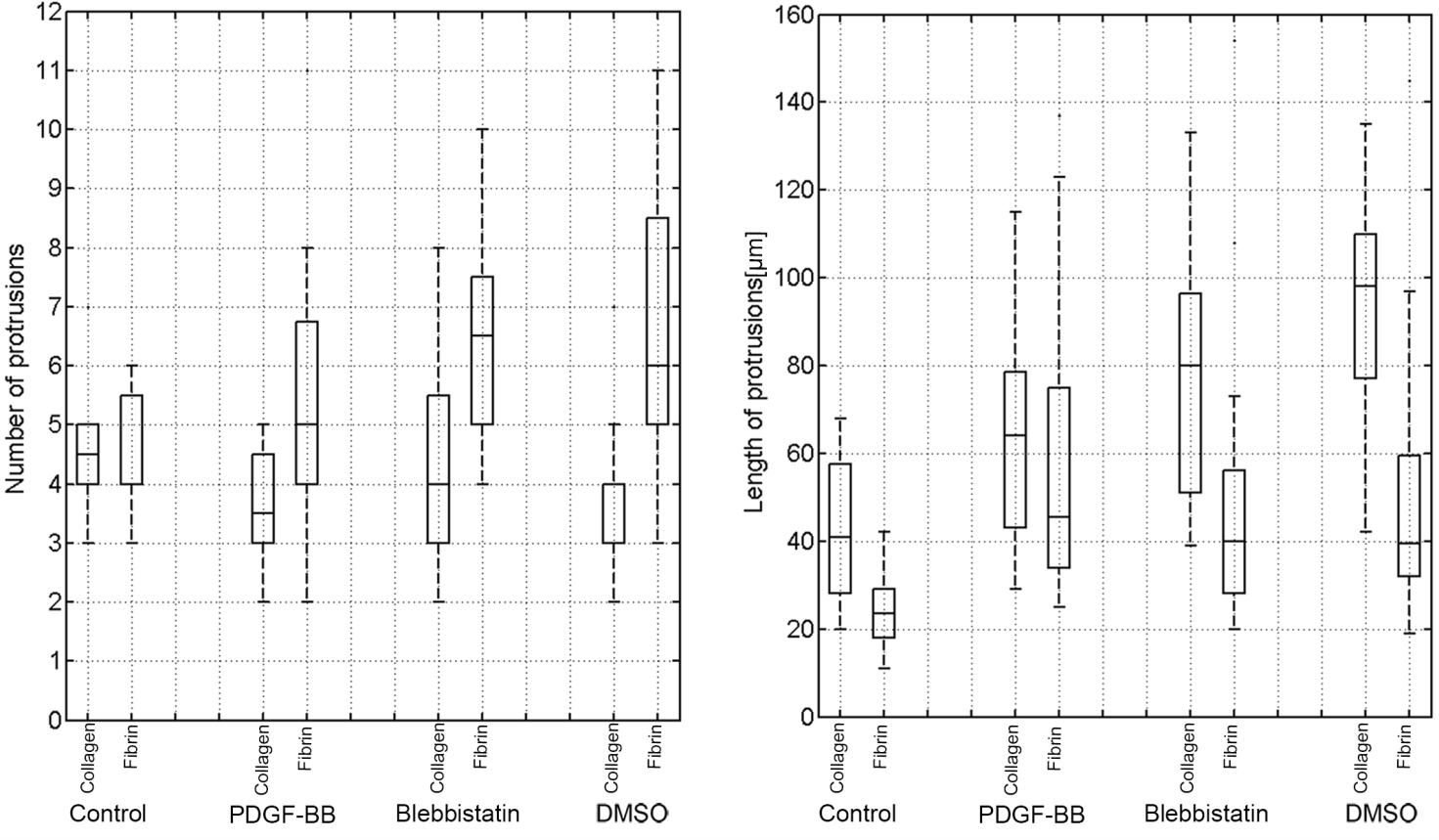


**Fig. S3.** Protrusion quantification. Measurements for the number (left) and length (right) of protrusions are represented in the boxplots for fibrin and collagen, for all the experimental conditions. The corresponding median and (1st | 3rd) quartile values, along with the statistical significances, are gathered in Table S3.

Supplementary tables

| **Table S1.** Median and (1^st^ \| 3^rd^) quartile values obtained from the migration directionality quantification.^†^ | | | |
| --- | --- | --- | --- |
|  | Collagen | Fibrin | |
| Control | 24.4 (-54.1 \| 77.6)^a^ | | -48.3 (-97.4 \| 69.9)^a^ |
| PDGF-BB | 81.0 (22.1 \| 99.2) | | 95.8 (79.6 \| 119.1) |
| Vehicle-control | 73.8 (35.1 \| 96.4) | | 90.9 (38.1 \| 111.6) |
| Blebbistatin | 76.3 ( 3.3\| 107.8) | | 51.4 (-67.9 \| 107.4) |
| ^†^ This values refer to Fig. 3, 6, 7, S1 and S2 and are expressed in degrees. ^a^Significant p-value < 10^-06^: control vs. PDGF-BB. | | | |

| **Table S2.** Median and (1^st^ \| 3^rd^) quartile values obtained from the migration speed quantification.^‡^ | | | | | | | | | | |  |
| --- | --- | --- | --- | --- | --- | --- | --- | --- | --- | --- | --- |
|  |  | | Collagen | | | | | | | | |
|  | Control | | | | | PDGF-BB | | Vehicle-control | | Blebbistatin | |
| Mean speed per zone | | Zone 1 | | 0.024 (0.013 \| 0.066)^*,a^ | 0.044 (0.016 \| 0.124)^b,α^ | | 0.054 (0.017 \| 0.141)^d,α^ | | 0.029 (0.014 \| 0.086)^α^ | | |
|  |  | Zone 2 | | 0.023 (0.012 \| 0.064)^*^ | 0.024 (0.013 \| 0.075)^c,β^ | | 0.031 (0.015 \| 0.085)^d^ | | 0.024 (0.011 \| 0.078) | | |
|  |  | Zone 3 | | 0.024 ( 0.013\| 0.067)^*,a^ | 0.030 (0.016 \| 0.085)^b^ | | 0.027 (0.013 \| 0.082) | | 0.023 (0.011 \| 0.064) | | |
| Effective speed per zone | | Zone 1 | | 0.008 (0.005 \| 0.013)^*,a^ | 0.066 (0.05 \| 0.078)^b,α^ | | 0.073 (0.041 \| 0.093)^α^ | | 0.038 (0.011 \| 0.047)^α^ | | |
|  |  | Zone 2 | | 0.007 ( 0.004\| 0.011)^*^ | 0.013 (0.006 \| 0.026) | | 0.020 (0.011 \| 0.046) | | 0.015 (0.005 \| 0.025) | | |
|  |  | Zone 3 | | 0.007 ( 0.004\| 0.009)^*^ | 0.009 (0.007 \| 0.02) | | 0.012 (0.01 \| 0.027) | | 0.008 (0.003 \| 0.014) | | |
|  |  | | Fibrin | | | | | | | | |
|  | Control | | | | | PDGF-BB | | Vehicle-control | | Blebbistatin | |
| Mean speed per zone | | Zone 1 | | 0.018 (0.01 \| 0.031)^a,α^ | 0.023 (0.012 \| 0.04)^c^ | | 0.026 (0.013 \| 0.058)^d^ | | 0.021 (0.01 \| 0.038)^α^ | | |
|  |  | Zone 2 | | 0.015 (0.008 \| 0.026)^a^ | 0.024 (0.011 \| 0.044)^b^ | | 0.025 (0.011 \| 0.049)^d^ | | 0.020 (0.008 \| 0.038) | | |
|  |  | Zone 3 | | 0.016 (0.008 \| 0.03)^a^ | 0.025 (0.012 \| 0.049)^b^ | | 0.026 (0.013 \| 0.053)^d^ | | 0.019 (0.008 \| 0.034) | | |
| Effective speed per zone | | Zone 1 | | 0.005 (0.001 \| 0.003)^a^ | 0.005 (0.004 \| 0.008)^α^ | | 0.005 (0.003 \| 0.009)^α^ | | 0.003 (0.001 \| 0.003)^α^ | | |
|  |  | Zone 2 | | 0.003 (0.001 \| 0.003) | 0.004 (0.002 \| 0.004) | | 0.005 (0.003 \| 0.006)^d^ | | 0.002 (0.001 \| 0.002) | | |
|  |  | Zone 3 | | 0.001 (0.001 \| 0.003) | 0.003 (0.001 \| 0.003) | | 0.005 (0.003 \| 0.005) | | 0.002 (0.001 \| 0.003) | | |
| ^‡^ This values refer to Fig. 3, 6, 7 and S2 and are expressed in μm·min^-1^. ^*, a, b, c, d^ Significant p-value < 10^-06^: ^*^ collagen vs. Fibrin; ^a^ control vs. PDGF-BB; ^b^ PDGF-BB vs. blebbistatin; ^c^ PDGF-BB vs. vehicle-control; ^d^ vehicle-control vs. blebbistatin. ^α, β^ Significant p-value < 0.001: ^α^ zone 1 vs. zone 2; ^β^ zone 2 vs. zone 3. | | | | | | | | | | |  |

| **Table S3.** Median and (1^st^ \| 3^rd^) quartile values obtained from the protrusion quantification.^δ^ | | | | | | | | |  |  |
| --- | --- | --- | --- | --- | --- | --- | --- | --- | --- | --- |
|  |  | | Collagen | |  | | Fibrin | | | |
|  | | Number of protrusions | | Length of protrusions (μm) | | Number of protrusions | | Length of protrusions (μm) | |  |
| Control | | 4.5 (4.0 \| 5.0) | | 41.0 (28.0 \| 57.5)^*^ | | 4.0 (4.0 \| 5.5) | | 23.5 (18.0 \| 29.0) | |  |
| PDGF-BB | | 3.5 (3.0 \| 4.5)^*,a^ | | 64.0 (43.0 \| 78.5)^*,a^ | | 5.0 (4.0 \| 6.7) | | 45.5 (34.0 \| 75.0)^a^ | |  |
| Blebbistatin | | 4.0 (3.0 \| 5.5) | | 80.0 (51.0 \| 96.5)^b^ | | 6.5 (5.0 \| 7.5)^b^ | | 40.0 (28.0 \| 56.0)^b^ | |  |
| Vehicle-control | | 4.0 (3.0 \| 4.0) | | 98.0 (77.0 \| 110.0) | | 6.0 (5.0 \| 8.5) | | 39.5 (32.0 \| 59.5) | |  |
| ^δ^ This values refer to Fig. S3. ^*, a, b^ Significant p-value < 0.05: ^*^ collagen vs. fibrin; ^a^ control vs. PDGF-BB; ^b^ control vs. blebbistatin. | | | | | | | | |  |  |

**Supplementary movies**

Sample movies of cells migrating in control samples, under PDGF-BB gradient and under PDGF-BB gradient including blebbistatin. Samples correspond to fibroblast 3D migration in collagen and fibrin gels:

- **SM1:** Control in collagen
- **SM2:** PDGF-BB gradient in collagen
- **SM3:** Blebbistatin containing PDGF-BB gradient in collagen
- **SM4:** Vehicle-control containing PDGF-BB gradient in collagen
- **SM5:** Control in fibrin
- **SM6:** PDGF-BB gradient in fibrin
- **SM7:** Blebbistatin containing PDGF-BB gradient in fibrin
- **SM8:** Vehicle-control containing PDGF-BB gradient in fibrin

The supplementary movies can be found at the zip file.
