# Supplementary figures and images for: Fibroblast Migration in 3D is Controlled by Haptotaxis in a Non-muscle Myosin II-Dependent Manner
Source: Ann Biomed Eng. 2015 May 27;43(12):3025–39. doi: 10.1007/s10439-015-1343-2 (PMC4623072; doi:10.1007/s10439-015-1343-2)

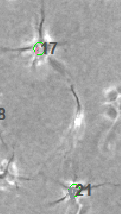

Supplement: Supplementary file 2 — Supplementary material 1 (ZIP 2506 kb) [file 10439_2015_1343_MOESM2_ESM.zip › Supplementary Movies/SM1_coll_control_thumbnail.tiff]

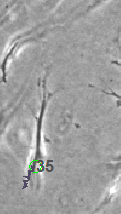

Supplement: Supplementary file 2 — Supplementary material 1 (ZIP 2506 kb) [file 10439_2015_1343_MOESM2_ESM.zip › Supplementary Movies/SM2_coll_PDGFBB_thumbnail.tif]

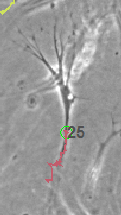

Supplement: Supplementary file 2 — Supplementary material 1 (ZIP 2506 kb) [file 10439_2015_1343_MOESM2_ESM.zip › Supplementary Movies/SM3_coll_blebbistatin_thumbnail.tif]

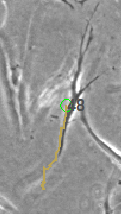

Supplement: Supplementary file 2 — Supplementary material 1 (ZIP 2506 kb) [file 10439_2015_1343_MOESM2_ESM.zip › Supplementary Movies/SM4_coll_dmso_thumbnail.tif]

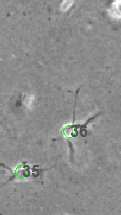

Supplement: Supplementary file 2 — Supplementary material 1 (ZIP 2506 kb) [file 10439_2015_1343_MOESM2_ESM.zip › Supplementary Movies/SM5_fibrin_control_thumbnail.tif]

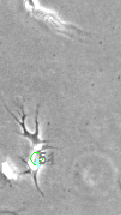

Supplement: Supplementary file 2 — Supplementary material 1 (ZIP 2506 kb) [file 10439_2015_1343_MOESM2_ESM.zip › Supplementary Movies/SM6_fibrin_PDGFBB_thumbnail.tif]

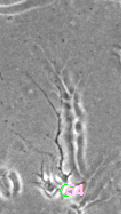

Supplement: Supplementary file 2 — Supplementary material 1 (ZIP 2506 kb) [file 10439_2015_1343_MOESM2_ESM.zip › Supplementary Movies/SM7_fibrin_blebbistatin_thumbnail.tif]

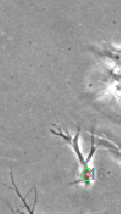

Supplement: Supplementary file 2 — Supplementary material 1 (ZIP 2506 kb) [file 10439_2015_1343_MOESM2_ESM.zip › Supplementary Movies/SM8_fibrin_dmso_thumbnail.tif]
